# Histomorphometric Evaluation of the Small Coronary Arteries in Rats Exposed to Industrial Noise

**DOI:** 10.3390/ijms160510095

**Published:** 2015-05-04

**Authors:** Ana Lousinha, Eduardo Antunes, Gonçalo Borrecho, Maria João Oliveira, José Brito, José Martins dos Santos

**Affiliations:** 1 Center for Interdisciplinary Research Egas Moniz (CIIEM), Health Sciences Institute, 2829-511 Monte de Caparica, Portugal; E-Mails: ejpantunes@sapo.pt (E.A.); gborrecho@gmail.com (G.B.); jaabrito@netcabo.pt (J.B.); jsantos@egasmoniz.edu.pt (J.M.S.); 2 Department of Anatomy, Abel Salazar Biomedical Sciences Institute, University of Porto, 4050-313 Porto, Portugal; E-Mail: mjoliveira@icbas.up.pt

**Keywords:** industrial noise, small coronary arteries, low-frequency noise

## Abstract

Morphological changes induced by industrial noise (IN) have been experimentally observed in several organs. Histological observations of the coronary arteries showed prominent perivascular tissue and fibrosis among IN-exposed rats. The effects on the small arteries are unknown. Objective: To evaluate the histomorphometric changes induced by IN on rat heart small arteries. Methods: Twenty Wistar rats exposed to IN during a maximum period of seven months and 20 age-matched controls were studied. Hearts were transversely sectioned from ventricular apex to atria and a mid-ventricular fragment was selected for analysis. The histological images were obtained with an optical microscope using 400× magnifications. A total of 634 arterial vessels (298 IN-exposed and 336 controls) were selected. The mean lumen-to-vessel wall (L/W) and mean vessel wall-to-perivascular tissue (W/P) ratios were calculated using image J software. Results: There were no differences between exposed and control animals in their L/W ratios (*p* = 0.687) and time variations in this ratio were non-significant (*p* = 0.110). In contrast, exposed animals showed lower W/P ratios than control animals (*p* < 0.001), with significant time variations (*p* = 0.004). Conclusions: Industrial noise induced an increase in the perivascular tissue of rat small coronary arteries, with significant development of periarterial fibrosis.

## 1. Introduction

Industrial noise (IN) is characterized by high intensity and a wide spectrum of wavelengths that includes low-frequency noise (LFN), this last characterized by large pressure amplitude ≥90 dB and low-frequency bands of ≤500 Hz [1]. Several morphological changes induced by IN and LFN have been experimentally observed in several tissues and organs [1,2,3,4,5,6,7].

We previously reported that coronary artery vessels showed prominent perivascular tissue and fibrotic development among IN-exposed rats [2] and also a significant fibrotic development in ventricular myocardium of rats exposed to LFN [3]. Considering the epidemiological evidence relating noise to ischemic heart disease and hypertension [8] and the effects of LFN on the extracellular matrix between the cardiomyocytes and around the cardiac vessels, which ultimately lead to myocardial stiffness and left ventricular disfunction and possibly to cardiac heart failure and arrhythmias [9,10], additional studies were performed. These showed a reduction of cardiac connexin 43 and a significant increase of cardiac collagen I and III after LFN exposure [4,5], reinforcing the hypothesis of an inducible morphological arrhythmogenic substrate.

The effects on the morphology of small arteries and arterioles in the rat heart are currently unknown, namely to what extent arteriolar wall thickening and perivascular fibrosis of the heart can be influenced by IN and LFN.

The aim of this study was to characterize the structural changes induced by IN on the rat heart small arteries.

## 2. Results

### 2.1. Data in Totum

#### 2.1.1. Lumen-to-Vessel Wall Ratio

We found no differences in the lumen-to-vessel wall (L/W) ratio between exposed and non-exposed animals. The histological evaluation did not show the presence of inflammatory cells in the two groups or modifications in the lumen or in the vessel wall.

The results of the histomorphometric analysis are shown in Table 1. The mean L/W ratio was 0.5560 and 0.5619, respectively, in groups A and B.

**Figure 1:**
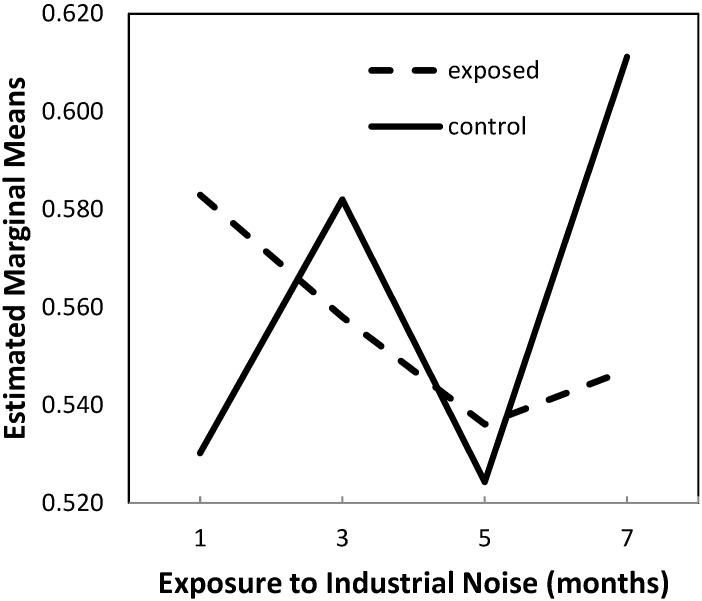
Estimated marginal means of lumen-to-vessel wall ratio.

The two-way ANOVA analysis of the data showed no differences between exposed and control animals in their L/W ratios (*p* = 0.687) and the time variations in this ratio were not significant (*p* = 0.110), as shown in Figure 1.

However, since a significant interaction between the independent variables was found in the model (*p* = 0.046), with observed power of 64.9%, we performed multiple comparisons by means of planned contrasts at different times that showed that, at month 7, there were significant differences between groups for this ratio *in totum*, with significantly decreased values in exposed animals compared to controls (*p* = 0.034).

Regarding differences between the groups in what concerns the time variations in this ratio, such differences were significant only for the changes observed from month 1 to 7 (*p* = 0.007), expressed as a 15.3% increase in the control group *versus* a 6.2% decrease in the exposed animals.

**Table 1:** Lumen-to-vessel wall ratio descriptive statistics in each group and overall, at the different times (IN = industrial noise).

Group	Exposure Time (Months)	Mean	Standard Deviation	*N*
IN exposed (group A)	1	0.5828933	0.03119288	5
	3	0.5579890	0.02053823	5
	5	0.5361110	0.04120586	5
	7	0.5471695	0.04270947	5
	Total	0.5560407	0.03675745	20
Control (group B)	1	0.5301545	0.04229613	5
	3	0.5820185	0.06370201	5
	5	0.5242906	0.06810608	5
	7	0.6111611	0.03569630	5
	Total	0.5619062	0.06211472	20

#### 2.1.2. Wall-to-Perivascular Tissue Ratio

**Figure 2:**
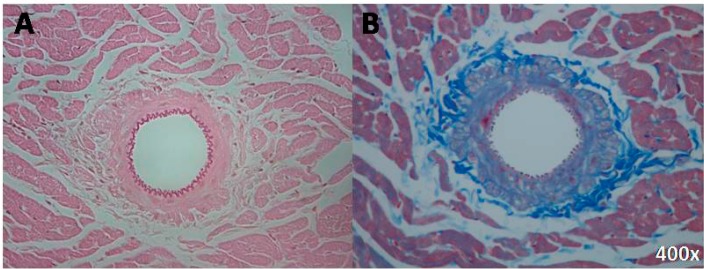
(**A**) Small coronary artery with a prominent perivascular tissue (IN group) (Hematoxylin-Eosin, 400×); (**B**) Small coronary artery with a prominent perivascular tissue, in the same group (Chromotrope-Aniline blue, 400×).

By contrast, the perivascular tissue was more prominent and seemed to show fibrosis in IN-exposed rats. Sections of the arteries are shown in Figure 2.

The mean wall-to-perivascular tissue (W/P) ratio was 0.4209 and 0.6373, respectively, in groups A and B (Table 2).

**Table 2:** Wall-to-perivascular tissue ratio descriptive statistics in each group and overall, at the different times (IN = industrial noise).

Group	Exposure Time (Months)	Mean	Standard Deviation	*N*
IN exposed (group A)	1	0.4322535	0.03050318	5
	3	0.3986722	0.02655070	5
	5	0.4234890	0.05262512	5
	7	0.4292097	0.04201934	5
	Total	0.4209061	0.03850858	20
Control (group B)	1	0.5849600	0.04896260	5
	3	0.6778928	0.01880381	5
	5	0.5745753	0.07750708	5
	7	0.7117764	0.01747083	5
	Total	0.6373011	0.07454984	20

**Figure 3:**
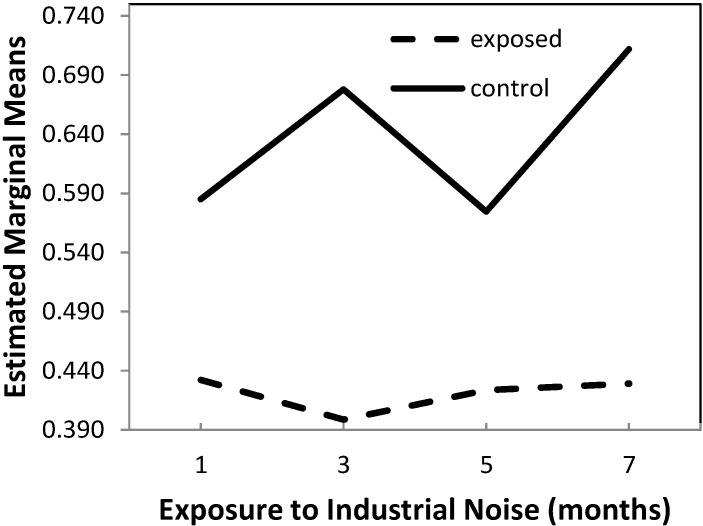
Estimated marginal means of wall-to-perivascular tissue ratio.

The results of the two-way ANOVA showed that there were significant effects on W/P ratio due to exposure (*p* < 0.001) and time variations (*p* = 0.004), which, however, are secondary, since a significant interaction between exposure and time exists (*p* = 0.001), as shown in Figure 3.

It is important to note that these effects do not seem to be due to chance, as the observed power is in excess of 90.7%.

In view of this significant interaction, planned contrasts were applied in the *post hoc* comparisons, which allow the following conclusions:

(1) At months 1, 3, 5 and 7, exposed animals have significantly lower W/P ratio than control animals (*p* < 0.001);

(2) From month 1 to 3, there is a 7.8% decrease in W/P ratio in exposed animals which differs significantly (*p* = 0.003) from the 15.9% increase in the same ratio observed in the control animals;

(3) From month 3 to 5, there is a 6.0% increase in W/P ratio in exposed animals, which differs significantly (*p* = 0.002) from the 15.2% decrease observed in the control animals;

(4) From month 5 to 7, there is an increase of 1.4% in W/P ratio in exposed animals, which is significantly lower (*p* = 0.002) than that of 23.8% observed in control animals.

### 2.2. Data Per Anatomic Region

#### 2.2.1. Lumen-to-Vessel Wall Ratio

The analysis of the data has shown that there were no significant effects of exposure to noise, duration of exposure and interaction between such factors on the L/W ratio in any the anatomic regions considered: right ventricle (RV), septum and left ventricle (LV).

#### 2.2.2. Wall-to-Perivascular Tissue Ratio

##### Right Ventricle

Significantly decreased W/P ratios were observed in the RV of exposed animal comparatively to controls (*p* > 0.001). No effect of duration (*p* = 0.111) or interaction between exposure and duration (*p* = 0.208) were observed in the same anatomic region.

##### Septum

The Two-way ANOVA approach to the data shows that there were significant effects on the W/P ratio in septum due to exposure (*p* < 0.001) and duration (*p* = 0.006), which were secondary, because of the significant interaction between exposure and duration (*p* = 0.002). It is important to note that these effects did not seem to be due to chance, as the observed power was in excess of 87.7%.

In view of the significant interaction, planned contrasts were applied in the *post hoc* comparisons, with the following conclusions:

(1) Exposed animals had significantly lower W/P ratio in septum than control animals, at all times (*p* ≤ 0.006);

(2) From month 1 to 3, there was a 13.8% decrease in W/P ratio in exposed animals which differed significantly (*p* = 0.003) from the 18.8% increase in the same ratio observed in the control animals;

(3) From month 3 to 5, there was a 13.2% increase in W/P ratio in exposed animals which differed significantly (*p* = 0.004) from the 16.7% decrease observed in the control animals;

(4) From month 5 to 7, there was an increase of 3.1% in W/P ratio in exposed animals which was significantly lower (*p* = 0.006) than that of 30.6% observed in control animals.

##### Left Ventricle

The Two-way ANOVA showed that there were significant effects on the W/P ratio in the LV due to exposure (*p* < 0.001) but not to duration (*p* = 0.282), and a significant interaction between exposure and duration was found (*p* < 0.001). Again, these effects did not seem to be due to chance, as the observed power was in excess of 98.3%.

Planned contrasts were applied in the *post hoc* comparisons, with the following conclusions:

(1) Exposed animals had significantly lower W/P ratio in LV than control animals, at all times (*p* < 0.001);

(2) From month 1 to 3, there was a 9.9% decrease in W/P ratio in exposed animals which differed significantly (*p* = 0.010) from the 12.9% increase in the same ratio observed in the control animals;

(3) From month 3 to 5, there was a 14.0% increase in W/P ratio in exposed animals which differed significantly (*p* = 0.006) from the 10.8% decrease observed in the control animals;

(4) From month 5 to 7, there was a decrease of 12.3% in W/P ratio in exposed animals which was significantly different (*p* = 0.006) from the increase of 20.3% observed in control animals.

## 3. Discussion

A number of animal studies found modifications in several tissues induced by low-frequency noise, characterized by abnormal deposition of collagen in the extracellular matrices [1,2,3,4,5,6,7].

In the present study we found an increase in the perivascular tissue around the small coronary arteries in rats exposed to IN. This is in agreement with the previous findings of our group, concerning the histomorphometric evaluation of the coronary arterial vessels in rats exposed to industrial noise [3]. In both studies we found the development of perivascular fibrosis in the absence of inflammatory cells and in the absence of obstructive coronary artery disease.

There were significant differences between exposed rats and controls concerning the mean vessel wall-to-perivascular tissue ratio, higher among the control group (*p* < 0.001). The effects of exposure time were observed in the whole population of rats and seemed to be independent of exposure to IN. The perivascular tissue was more exuberant at three months of IN exposure, with a gradual reduction observed until seven months. It is important to note that, despite this gradual reduction, it remained increased among the IN-exposed rats compared to control at all times.

The analysis of data per anatomic region showed no particular differences between the right ventricle, septum and left ventricle, confirming that the influence of industrial noise over the heart is global.

Regarding the results concerning the ratio lumen-to-vessel wall between exposed and non-exposed animals, no significant differences were found until seven months of exposure to IN. At this point, an increased wall thickeness of intramyocardial small coronary arteries was observed in exposed rats*,* as compared to controls (*p* = 0.034). Previous studies performed on large vessels found the same type of modifications in the vessel wall [6]. In contrast, such differences were not observed in the coronary arterial vessels [3]. In this case, we speculated that a lower susceptibility of coronary arterial vessels to IN damage may occur, as suggested by the absence of the internal elastic lamina disruption and the fact that no proliferation of smooth muscle cells was observed in the intima up to seven months of exposure. Taking into consideration our recent results, we may further speculate that industrial noise effects on wall thickening require longer times of exposure and that small coronary arteries are the first to be affected.

A previous review of epidemiological studies concerning environmental noise exposure (including road and aircraft noise sources) and cardiovascular risk reported increasing evidence relating noise and hypertension and ischemic heart disease [8], making pertinent to investigate the effects of IN on the heart.

Thus far, we documented that coronary artery vessels showed prominent perivascular tissue and fibrotic development among IN-exposed rats [3] and also a significant fibrotic development in ventricular myocardium of rats exposed to LFN [2]. Considering that these structural changes ultimately lead to myocardial stiffness and left ventricular disfunction and possibly to cardiac heart failure and arrhythmias [9,10], additional studies were performed. These showed a reduction of cardiac connexin 43 and a significant increase of cardiac collagen I and III after LFN exposure [4,5], reinforcing the hypothesis of an inducible morphological arrhythmogenic substrate. The present study allowed the documentation of structural changes induced by industrial noise on the rat heart small arteries, suggesting a general influence of industrial noise over the heart.

It remains to understand the fibrotic proliferation mechanism behind the IN. A strong possibility, taking into consideration the dynamic interactions between fibroblasts and the extracellular matrix [11], could be the occurrence of an abnormal biological fibroblastic response induced by IN through a mecanotransduction process [12]. In addition, we could speculate that IN can induce fibrosis through the loss of regulation between profibrotic and antifibrotic molecules, carried out by mechanical and neurohumoral factors [13].

The changes in the structure of coronary resistance vessels, with quantitative characterization of small coronary arteries and arterioles in the myocardium, have been extensively studied under several experimental conditions and were already extended to humans. Cardiac hypertrophy in hypertension, with an increase in left ventricular mass, is characterised by increased wall thickness in arterioles and small arteries, increased lumen to wall ratio, and decreased number of capillary profiles per arteriole in cross section [14]. Volume overload-induced cardiac hypertrophy is characterized by normal coronary reserve and maximal flow and there is evidence that both arteriolar and capillary growth is proportional to the magnitude of hypertrophy [15]. Pathologic findings have described diabetic cardiomyopathy as a microvascular disease and previous studies have demonstrated that structural changes of coronary microvessels in diabetes include thickening of the vascular wall, perivascular fibrosis, capillary aneurysms, and decrease in capillary density [16,17]. Several structural abnormalities of the heart are present in chronic renal failure, including arteriolar thickening [18], reduced capillary density [19], and interstitial fibrosis [20]. These findings contribute to myocardial ischemia, left ventricular wall stiffness, diastolic dysfunction, and arrhythmogenicity in patients with renal failure [21].

It will be our challenge in the future to understand the clinical impact on cardiac diseases among humans exposed to industrial and low-frequency noise.

We are aware of several limitations of the study. We admit that interpretation of the results must be done cautiously because, for logistical reasons, the number of animals per group was limited. Additionally, small coronary arteries in biopsy samples were partially crushed during the procedure, which might have affected the quantitative analyses of lumen area and perivascular tissue. Furthermore, we acknowledge that at the present time there is not a well-defined morphological cardiac model induced by industrial noise. Thus far, we have limited our observations to the structural modifications induced by industrial noise or by low-frequency noise in the myocardium of rat heart and can only extrapolate these observations to humans, with all the existing limitations. The experimental conditions tried to simulate the schedule of industrial plant workers, characterized by 8 hours/day, 5 days/week of exposure to industrial noise. Once again, we reinforce the need of clinical investigations concerning the effects of industrial and low-frequency noise on the heart.

## 4. Experimental Section

Forty adult Wistar rats were studied. The animals were treated in accordance with the EU Commission on Animal Protection for Experimental and Scientific Purposes (86/609/EEC) and with the Portuguese legislation for the same purpose (Decree-Law No. 197/96). All the animals were kept in cages, fed standard rat food, and had free access to water. Twenty animals (Group A) were exposed to IN for a period of one to seven months, in an occupationally simulated schedule (8 hours/day, 5 days/week, and weekends in silence). The remaining 20 rats were used as age-matched controls (Group B) and were kept in a silent environment. Each group was divided into four subgroups with five rats and sacrificed after 1, 3, 5 and 7 months.

The sound signal was emitted by an analog noise generator, amplified and frequency filtered. The noise level was the same as previously reported, characterized by a wide spectrum of frequencies but with an important component under 500 Hz [11].

The hearts were fixed in 10% buffered formalin, sectioned transversely from the ventricular apex to the atria and prepared for histological observation using hematoxylin eosin and chromotrope-aniline blue (CAB) staining. The mid-ventricular fragment from each heart was selected for the study. The histological images were acquired with an optical microscope using 400× magnification.

A total of 634 arterial vessels were selected (298 in group A and 336 in group B). Data were analyzed using the computer image analysis image J software (National Institutes of Health, Bethesda, MA, USA). The caliber of the arterial vessels, the thickness of the walls and the perivascular tissue dimension were measured and the mean lumen-to-vessel wall (L/W) and mean vessel wall-to-perivascular tissue (W/P) ratios were calculated.

### Statistical Analysis

The morphometric data are presented as mean ± standard deviation. A two-way ANOVA model was applied in order to compare animals exposed to noise with non-exposed age-matched controls, in what concerns time variations (at months 1, 3, 5 and 7) in the L/W and W/P ratios assessed in the cardiac muscle, *in totum* and per anatomic region (right ventricle, septum, and left ventricle). The appropriateness of the ANOVA model is justified by the fact that the 8 subgroups defined by the factors levels are of equal size (*n* = 5), which warrants the robustness of the F ratio statistics under potential heterogeneous variances. A *p* value <0.05 was considered statistically significant.

## 5. Conclusions

In conclusion, industrial noise induced an increase in the perivascular tissue of rat small coronary arteries, with significant development of periarterial fibrosis.
